# Relationship of quercetin intake and oxidative stress in persistent COVID

**DOI:** 10.3389/fnut.2023.1278039

**Published:** 2024-01-08

**Authors:** Diana Matías-Pérez, Carolina Antonio-Estrada, Araceli Guerra-Martínez, Karen Seydel García-Melo, Emilio Hernández-Bautista, Iván Antonio García-Montalvo

**Affiliations:** ^1^Division of Graduate Studies and Research, Tecnológico Nacional de México/Instituto Tecnológico de Oaxaca, Oaxaca, Mexico; ^2^Department of Chemical Engineering, Tecnológico Nacional de México/Instituto Tecnológico de Oaxaca, Oaxaca, Mexico

**Keywords:** COVID-19, ROS, flavonoids, quercetin, post-pandemia

## Introduction

**Figure 1:**
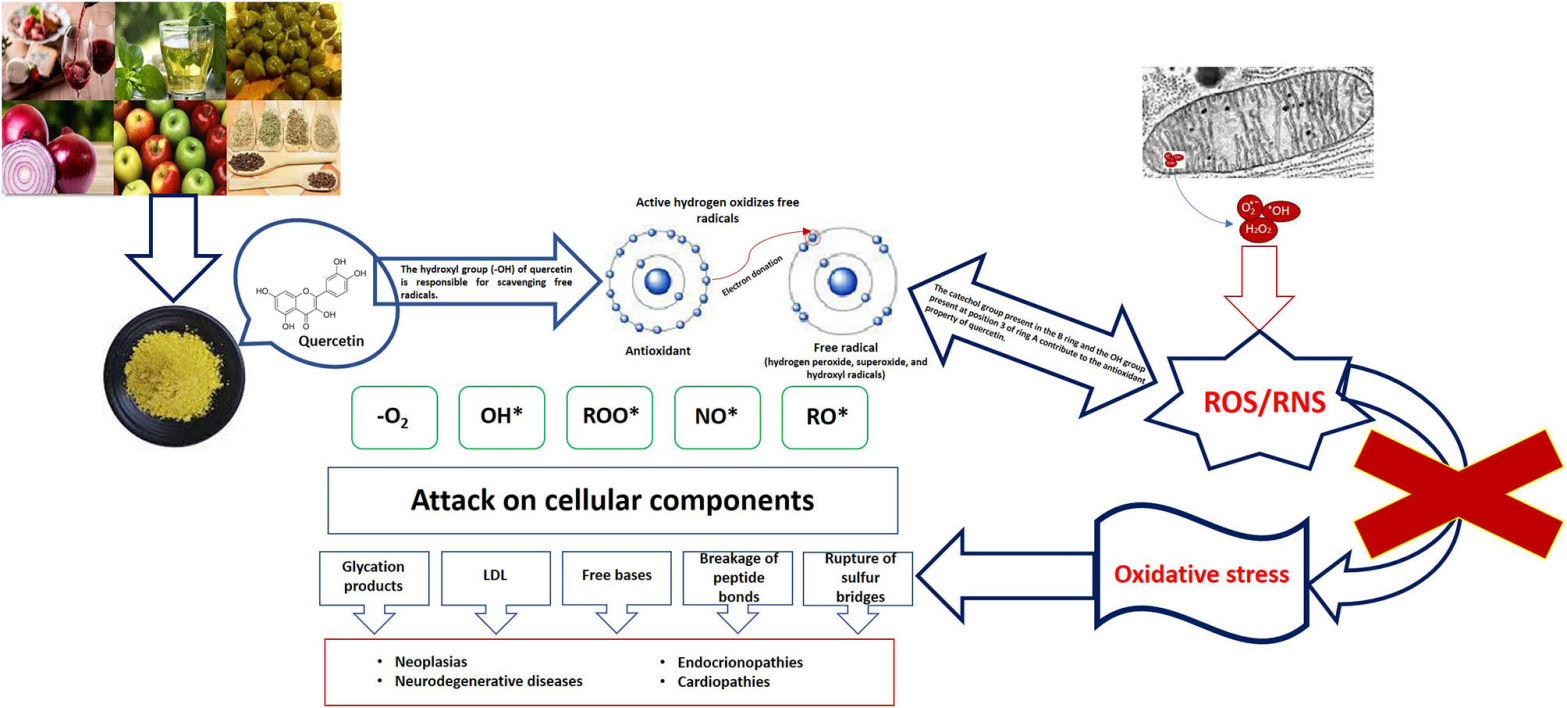
Oxidative damage and role of quercetin.

The health emergency resulting from the COVID-19 pandemic has come to an end, as announced by the World Health Organization (WHO) on 5 May this year. Three years after the onset of this health emergency, the WHO said that although the emergency phase is over, the pandemic is not over. However, the sequelae caused by the SARS-CoV-2 virus continue, the fact that patients experience symptoms after recovery from acute infection is not unexpected and is associated with an increased risk of post-infectious sequelae, known as persistent COVID or post-acute sequelae of COVID-19. These sequelae may present with various long-lasting symptoms in the absence of active infection, these symptoms include: the presence of musculoskeletal pain, aging fatigue, mood disturbance, and neurocognitive difficulties (1–4). Currently, persistent COVID does not have effective validated treatments; it is a multifactorial pathology, in which the literature mentions some causes, such as persistence of SARS-CoV-2 reservoirs in body tissues (5, 6); immune dysregulation with or without reactivation of the underlying pathogens (7, 8), the emergence of the Epstein-Barr virus or the human herpesvirus-6 (9–12), microbiota conditions derived from the SARS-CoV-2 attack (6, 13–15), autoimmunity (6, 16–18), microvascular blood coagulation with endothelial dysfunction (6, 19–21) as well as dysfunctional signaling in the brainstem and/or vagus nerve (6, 22, 23), this combined with risk factors such as type 2 diabetes mellitus, sex (mainly female), ethnicity (of Latino origin), socioeconomic factors (low income), exposure to COVID-19 reinfection, the presence of specific antibodies, connective tissue disorders, and attention deficit hyperactivity disorder, however, one third of people with persistent COVID have no preexisting conditions identified (23). Redox abnormalities that occur in persistent COVID are due to functional instability of the mitochondria in addition to alterations in the oxidative stress pathways (24, 25), not leaving aside the presence of a chronic hyperinflammatory condition (26). A lifestyle that involves a lower intake of ultraprocessed and processed foods, daily exercise, and the inclusion of nutritional supplements rich in carotenoids, omega 3, or flavonoids can help in the treatment of persistent COVID. Specifically, flavonoids are a group of natural polyphenolic substances naturally present in different flowers, fruits, vegetables, seeds, and beverages derived from plants such as tea and red wine, which are considered responsible for their characteristic color (27). Quercetin belongs to the classification of flavonoids, and its beneficial functions are associated with its chemical structure, which gives it antioxidant properties. Quercetin neutralizes free radicals such as superoxide anions, nitric oxide, and peroxynitrites (see Figure 1) (28, 29). It can inhibit enzymes such as xanthine oxidase, lipoxygenase, and NADPH oxidase, preventing cell death; in addition, it increases the production of endogenous antioxidants (30, 31). Derived from the antioxidant, anti-inflammatory, hypoglycemic and hypolipidemic properties of Quercetin, it is a good alternative for the treatment of Type 2 diabetes mellitus (T2DM). By reducing the concentration of glucose levels in the blood, it preserves the function of islet cells, increases the number of pancreatic β-cells, reduces dyslipidemia, increases insulin level and reduces damage from oxidative stress (increases the activity of catalase and heme oxygenase enzymes) (32, 33). Evidence of quercetin administration in diabetic mice for 10 days at 10–15 mg/kg shows a reduction in peripheral blood glucose and triglyceride levels, as well as increased enzyme activity of hexokinase and glucokinase (34). Mahadev et al. (35) recommends that the consumption of Quercetin (15–100 mg/kg) should be for a period of 14–70 days to be considered as a potential alternative in the treatment of T2DM. Quercetin has been shown to bring health benefits with respect to age-related diseases, such as neurodegenerative diseases, age-related macular degeneration, bone metabolism diseases, cardiovascular diseases, cancer, as well as having anti-inflammatory and hepatoprotective functions (33, 36–42).

## Oxidative stress in persistent COVID

The oxidative stress that occurs in COVID-19 is related to the cytokine storm, the coagulation mechanism, and the exacerbation of hypoxia; elevated inflammatory and oxidative state in the pathology triggers mitochondrial oxidative stress and dysfunction, which contributes to dysbiosis or imbalance of the balance of the intestinal microbiota, increasing the inflammatory and oxidative response (43–45). Viral infections alter the antioxidant mechanisms, generating a pro-oxidant action, leading to an unbalanced oxidative-antioxidant state and consequent oxidative cell damage (45). Viral infection through SARS-CoV-2 allows the innate immune system to identify infection through pattern recognition receptors (PRRs); these involve toll-like receptors (TLRs), which trigger a pro-oxidant response of macrophages, resulting in activation of the TNF-α and NADPH-oxidase in leukocytes, as well as mediating the production of reactive oxygen species (ROS) (46). In subjects with persistent COVID, the hyperinflammatory state involves systemic disturbances in the host, such as iron dysregulation that manifests itself as hyperferritinemia associated with disease severity, which induces ROS production promoting oxidative stress (47). The enzyme nitric oxide synthase induces in neutrophils the production of oxygen free radicals capable of combining with nitric oxide (NO) to generate the compound peroxynitrite; neutrophilia generates an excess of ROS that exacerbates the host's immunopathological response, resulting in more severe disease (46, 47).

## Quercetin and persistent COVID

Quercetin has antioxidant, anti-inflammatory, anticarcinogenic, and immunoprotective functions because it promotes mitochondrial biogenesis, inhibits lipid peroxidation, inhibits capillary permeability, and inhibits platelet aggregation; in viral infections inhibits the binding of viral capsid proteins and controls the production of proteases and polymerases (48, 49). Specifically, SARS-CoV-2 inhibits enzymes involved in virus replication (49–51). Quercetin alters the expression of 30% of genes encoding SARS-CoV-2 target proteins in human cells, potentially interfering with the activities of 85% of SARS-CoV-2 proteins (52). Quercetin inhibits protein disulfide isomerase (PDI), which is the enzyme involved in platelet-mediated thrombin formation, thus improving the coagulation abnormalities that can be found in subjects with persistent COVID (53). Quercetin has been shown to be an effective inhibitor against several viruses *in vitro*, such as rhinovirus serotypes, echovirus (type 7, 11, 12 and 19), coxsackievirus (A21 and B1), poliovirus (type 1 Sabin) at a minimum inhibitory concentration of 0.03–0.5 μg/ml in Hela or WI-38 cells (54). Quercetin significantly reduces RNA and DNA replication in herpes simplex virus 1 (HSV-1), parainfluenza type 3, polio type 1, showing anti-infectious and anti-replicative properties (55). Studies have shown that it inhibits HeLa cell replication inoculated with cytomegalovirus (CMV) at a mean inhibitory concentration of 3.2 ± 0.8 μM (56); work has been done on the replication of dengue virus type 2 (DENV-2) in Vero cells and in which Quercetin inhibited at a mean concentration of 35.7 μg/ml, causing a DENV-2 RNA reduction of 67%. This is attributed to the ability to block viral entry or inhibit viral replication enzymes, such as viral polymerases (57, 58). A mixture of Quercetin with Vitamin C can disrupt virus entry, replication, enzymatic activity, and assembly, while seeking to strengthen the immune response by promoting early IFN production, modulating interleukins, promoting T-cell maturation and phagocytic activity (58). Quercetin seeks to inhibit SARS 3CL protease by binding to its GLN189 site, which is expressed similarly in SARS-CoV-2, thus providing the mechanism for its experimental clinical use, in addition to its own immunomodulatory and anti-inflammatory actions (59, 60). It is important to mention that Quercetin can be found in foods and beverages of vegetable origin (grapes, red onion, broccoli, grapefruit, apples, cherries, green tea and red wine), it represents 60%−75% of the total flavonols consumed, its half-life in humans has been estimated to be 31–50 h, with a peak plasma concentration half an hour after consumption and another 8 h after 100 mg ingestion (61). However, the amount of Quercetin contained in the plant is conditioned by several factors: (a) the part of the plant in which it is found, the majority in the external areas; (b) the time of the year in which it develops, in summer and with greater exposure to the sun there will be more flavonoids, warm climates favor the synthesis of Quercetin; (c) the more mature the fruit, the higher the Quercetin content; (d) the process of preparation and processing of the food also has an influence, the fact that cooking these plants can reduce the amount of Quercetin they possess, in addition, it is also lost by removing the skin of the fruit or vegetable. The low bioavailability of Quercetin and its poor solubility are limitations to its use leading to the reduction of the antioxidant power it possesses; restricted transport across biological barriers and transient retention are challenges to overcome, and an alternative to the above is the use of nanotherapy. The use of conjugates and nanocarriers based on different materials is an option to consider for Quercetin; these nanocarriers have been used with natural compounds such as Ginkgo Biloba as well as targeted drugs for neurodegenerative diseases (62–64); these nanoparticles can be organic or inorganic, organic materials (liposomes, micelles, and polymeric nanoparticles) stand out for being compatible and degradable in their entirety. Inorganic nanoparticles (iron oxide nanoparticles, gold nanoparticles, and silica nanoparticles) have smaller size, stability, higher permeability, and a controlled release period (65–67).

## Conclusion

In conclusion, we can say that the current prevalence of persistent COVID symptoms is striking, due to oxidative stress that plays an important role in the progression of this pathology. The use of antioxidants for the elimination of free radicals is an appropriate strategy, considering that in recent decades the intake of substances with antioxidant properties, such as quercetin, has increased; this increase in consumption can be achieved through diet or using food supplements with higher concentrations of the flavonoid than those naturally occurring in food. It is worth mentioning the role played by quercetin in the reduction of viral load, decrease in the release of pro-inflammatory cytokines, reduction of ROS, decrease in mucus production, with respect to the above we can say that it increases the resistance of the respiratory tract; moreover, quercetin has not yet shown any harmful effects in humans at a maximum dose of 1,500 mg per day.

## Author contributions

DM-P: Investigation, Writing—original draft. CA-E: Investigation, Writing—original draft, Formal analysis, Methodology. AG-M: Formal analysis, Investigation, Methodology, Writing—original draft. KG-M: Formal analysis, Investigation, Methodology, Writing—original draft. EH-B: Investigation, Methodology, Writing—review & editing. IG-M: Investigation, Conceptualization, Supervision, Writing—original draft.
